# A Meta-Analysis of the Prevalence of Wheat Allergy Worldwide

**DOI:** 10.3390/nu15071564

**Published:** 2023-03-23

**Authors:** Wenfeng Liu, Yong Wu, Jian Wang, Zhongliang Wang, Jinyan Gao, Juanli Yuan, Hongbing Chen

**Affiliations:** 1 State Key Laboratory of Food Science and Technology, Nanchang University, Nanchang 330047, China; 2 School of Food Science and Technology, Nanchang University, Nanchang 330031, China; 3 Sino-German Joint Research Institute, Nanchang University, Nanchang 330047, China; 4 Jiangxi Province Key Laboratory of Food Allergy, Nanchang University, Nanchang 330047, China; 5 School of Pharmaceutical Sciences, Nanchang University, Nanchang 330006, China

**Keywords:** wheat allergy, prevalence, meta-analysis, worldwide

## Abstract

Wheat allergy is a primary disease of food allergy, and its global prevalence is unclear. This study aimed to characterize the latest worldwide prevalence of wheat allergy based on five different diagnostic methods. Study searches were conducted in Web of Science, PubMed, Ovid LWW, and Cochrane database, with a time limit of 1 January 2007 to 1 September 2022. The review and screening of the articles was undertaken by two independent reviewers. The statistical analysis was conducted by R. A total of 56 articles were finally included. The prevalence of wheat allergy was 0.63% (95% CI: 0.43–0.87%) for self-reported, 0.70% (95% CI: 0.18–1.22%) for self-reported physician-diagnosed, 0.22% (95%CI: 0.07–0.65%) for skin prick test positive, 0.97% (95% CI: 0.43–2.20%) for specific immunoglobulin E positive, and 0.04% (95% CI: 0–0.16%) for food challenge. However, food challenge can be largely subjective, and the results were only based two countries, so the prevalence of wheat allergy confirmed by food challenge may be not entirely trustworthy. In conclusion, investigating the prevalence of wheat allergy in the real world as accurately as possible will contribute to the prevention, management, and risk assessment of wheat allergy.

## 1. Introduction

Wheat is recognized as a common trigger of immune-mediated food allergy, which has become a public health and food safety issue of global importance, posing a substantial financial and health burden [1,2]. The proteins in wheat that can cause allergic reactions, including those related to gliadin, glutenin, albumin, and globulin. Moreover, omega-5 and gamma gliadin are major allergens in adults with wheat allergy in Thailand [3]. Alpha-amylase/trypsin inhibitor family members exhibit strong IgE reactivity in wheat flour [4], and omega-5 gliadin and high molecular weight gluten are major allergens associated with wheat-dependent exercise-induced anaphylaxis (WDEIA) [5]. Wheat allergy affects people in many countries of the world, seriously damaging people’s health. In Europe, wheat was the most common food trigger of anaphylaxis in adults, and wheat anaphylaxis was more prevalent in central Europe than in southern Europe [6]. In China, a 14-year retrospective study analyzed 907 individuals who were diagnosed with anaphylaxis or severe allergic reaction, showing that wheat allergens were the main trigger of food-induced anaphylaxis in adults and children [7]. Wheat has attracted massive global attention as inducing mild to severe adverse reactions, and it is essential to understand the hazard, especially the prevalence of wheat allergy.

Immunologic reactions to wheat can be divided into IgE-mediated reactions and non-IgE-mediated reactions [8]. Patients with wheat allergy may suffer from an adverse reaction when they are exposed to wheat and its products, such as atopic dermatitis, urticaria and gastrointestinal symptoms, etc. Immediate response is one of the characteristics of IgE-mediated wheat adverse reaction, and the onset of symptoms typically does not exceed 2 h after exposure to wheat and its products [9]. Meanwhile, non-IgE-mediated wheat allergy is dependent on specific immune cell-mediated immune responses, and the main clinical characteristics include eosinophilic esophagitis, eosinophilic gastroenteritis, or eosinophilic colitis.

Patients with wheat allergies can be effectively protected by strict avoidance of wheat allergens in addition to immunotherapy. Moreover, implementing quantitative risk assessment for allergens and mandatory allergen labelling could help patients with wheat allergy avoid exposure to wheat allergens. In 2020, WHO/FAO convened 20 of the world’s leading experts and academics, as well as 10 resource persons, to form an ad hoc joint expert group to review the existing food allergy list based on a risk assessment approach [10]. The prevalence (e.g., the proportion of a defined population known to have experienced an immune-mediated adverse reaction to food), potency (e.g., the amount of the total proteins from the food/ingredient required to cause objective symptoms in a specified proportion), and severity (e.g., frequency or proportion of severe objective reactions to a food/ingredient such as anaphylaxis) of food allergens were the criteria for ad hoc joint WHO/FAO expert consultation to identify priority allergens. Clinically, the diagnosis of wheat allergy was based on the skin prick test (SPT), serological tests (sIgE), and food challenge (FC) combined with clinical history [11]. Importantly, prevalence is a key criterion for allergen risk assessment. Different clinical diagnostic methods would provide different prevalence results. Investigating the point prevalence of different diagnostic methods and insight into the prevalence could benefit the prevention and management of wheat allergy. In fact, the point prevalence of wheat allergy on a global scale is unavailable. Therefore, the aim of this study is to synthesize high-quality prevalence data of wheat allergy based on the literature collection, screening, and data processing to provide a comprehensive analysis of the prevalence of the global population and to analyze the difference among age groups, countries with different economic conditions, and region groups.

## 2. Materials and Methods

### 2.1. Protocol and Registration

The protocol of this meta-analysis was registered with the International Prospective Register of Systematic Reviews (PROSPERO; http://www.crd.york.ac.uk/prospero, accessed on 17 May 2022, CRD42022318766).

### 2.2. Search Strategy

The search strategy mainly referred to Roberto J. Rona [12], with modification. Briefly, the search strategy consists of three groups of terms: (1) “wheat” or “gluten” or “food”; (2) “allergy” or “anaphylaxis” or “hypersensitivity” or “sensitization” or “immunoglobulin E” or “skin prick test” or “challenge” or “provocation”; and (3) “prevalence” or “epidemiology” or “incidence”. The following databases were used: Web of Science, PubMed, Ovid LWW, and Cochrane. For the search results, the following study designs were included: cohort studies, cross-sectional studies, case control studies, and routine health care. Certain types of studies were excluded from this review, including reviews, systematic reviews and meta-analysis, case studies, conference abstracts, case series, and animal studies. All eligible articles must have been published between 1 January 2007 and 1 September 2022. The language of articles was restricted to Chinese and English.

### 2.3. Definition

In this study, we analyzed the wheat allergy prevalence based on five diagnostic methods. The related definitions are as follows.

Self-reported wheat allergy (SR wheat allergy): self-reported wheat allergy based on questionnaire survey.

Self-reported physician diagnosed wheat allergy (SRPD wheat allergy): self-reported wheat-related adverse reaction diagnosed by physicians.

SPT positive: individuals with reactive SPTs to wheat allergens, and wheal size exceeded 3 mm at least.

Wheat allergy for SPT positive: SPT positive with clinical history.

sIgE positive: positive serology to wheat allergens, and the value of cut off exceeded 0.35 kUA/L at least.

Wheat allergy for sIgE positive: sIgE-positive with clinical history.

Wheat allergy confirmed by FC: positive reaction (objective immediate symptoms or subjective symptoms in some cases) to wheat and its product in any kind of oral food challenge.

The investigated population were stratified by age into children (0–17 years old), adults (≥18 years old), and other (the age range spanned children and adults or undefined age range). The subgroup of developed economies, developing economies, and economies in transition were set up in this study according to the basic economic condition [13]. Additionally, three timespans of 2007–2011 (timespan-1), 2012–2016 (timespan-2), and 2017–2022 (timespan-3) were set up. Moreover, the whole world regions could be divided into six regions, which were the African Region, South-East Asia Region, Eastern Mediterranean Region, Region of the Americas, European Region, and West Pacific Region according to the principles of the World Health Organization (WHO) [14].

### 2.4. Study Selection and Data Extraction

Two independent reviewers evaluated the titles and abstracts of the retrieved articles and categorized them into included, excluded, and unsure. Moreover, two independent reviewers checked the full texts of the unsure articles above and recategorized. Any discrepancies were resolved by a consensus or arbitrated by a third reviewer. Data extraction of included articles was performed by two independent reviewers, and any inconsistences were checked by a third reviewer. The first author, published year, study design, study period, country, age of patients, and outcome reported of the articles were extracted.

### 2.5. Risk of Bias Assessment

The risk of bias assessment was conducted by two independent reviewers using the Joanna Briggs Institute (JBI) Critical Appraisal Checklist for Studies Reporting Prevalence Data [15] (Table S1). Any disagreement that arose between the reviewers were resolved through discussion, or with another reviewer. There were nine questions in the checklist, and each question should be answered by “yes”, “no”, or “unclear”. The answer of “yes” was scored “1”, and the answer of “no” or “unclear” was scored 0. The high-quality study was defined as the score of 8–9, the moderate quality study was defined as the score of 5–7, and the low-quality study was defined as the score of 0–4. The low-quality studies were excluded from meta-analysis. Any discrepancies were resolved by consensus or arbitrated by a third reviewer.

### 2.6. Meta-Analysis of the Prevalence of Wheat Allergy

The meta-analysis was performed by the R program with version of 4.2.0 [16]. Briefly, after loading the “meta” package in R and reading the data by the ‘read.csv’ command, the ‘rate’ was defined as the proportion of the allergic population, which was determined by dividing the number of individuals described as wheat allergy by the total number of people surveyed. The “rate” of each study was categorized based on diagnostic methods and comprised five datasets. The Shapiro-Wilk test was applied to determine whether each dataset followed a normal distribution. If the dataset met the normal distribution, the meta-analysis would be performed directly, if not, the “rate” was transformed by “log”, “logit”, or “arcsin”, and then the meta-analysis was performed. The prevalence of wheat allergy was analyzed based on different diagnostic methods, and the results were presented in a forest plot. For each diagnostic method, a Chi-square (χ^2^) test was used to analyze whether age, basic economic conditions, and geographical location that would have an effect on prevalence. The heterogeneity of studies was assessed by the I-squared (*I^2^*) method. The option of effects model for clinical and methodological studies to assess the point size frequency and 95% confidence interval (95% CI) of wheat allergy depended on the heterogeneity. If the *I^2^* ≤ 50% and *p* ≥ 0.05, the common effect model was applied, and sensitivity analysis was performed. If the *I^2^* > 50% and *p* < 0.05, the random effect model was applied, and sensitivity analysis was performed. The publication bias was conducted by Egger’s test, since the data itself or the transformed data fit the normal distribution. The trim-and-fill method was applied in this study.

## 3. Results

### 3.1. Study Selection

**Figure 1:**
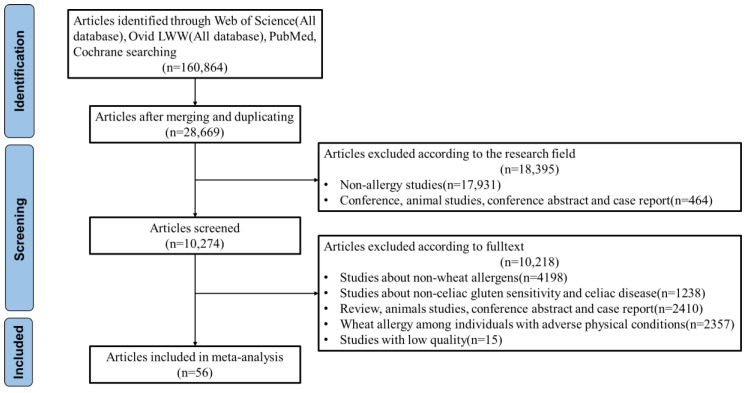
Flowchart for studies on the prevalence of wheat allergy, 1 January 2007–1 September 2022.

Figure 1 showed the flowchart for the study screening. Searching through the four databases above, 28,669 articles were collected after removal of duplicates. Subsequently, the titles and abstracts of these articles were reviewed, generating 10,274 articles. Moreover, full text of the remaining articles was reviewed, where the studies about non-wheat allergens, non-Celiac gluten sensitivity, and Celiac disease were excluded. Wheat allergy among individuals with adverse physical conditions and case studies, as well as conference abstracts about wheat allergy, were also excluded. Accordingly, 56 articles were finally included for meta-analysis.

### 3.2. Study Characteristics

About 56 studies were included in meta-analysis, and 29 studies were conducted in regions of developed economies, and one study was a multicenter study involving regions of developing economic condition and economies in transition, while 25 studies were conducted in regions of developing economies, and one was conducted in the region of economies in transition. Moreover, the statistics based on diagnostic methods among the included studies indicated that 28 studies identified wheat allergy through SR only, one study identified wheat allergy through SRPD only, and six studies investigated both SR and SRPD. Seven studies identified wheat allergy only through SPT, nine studies only through sIgE, and two studies investigated wheat allergy either for SPT positive or sIgE positive. Additionally, three studies identified wheat allergy through FC, and one of them utilized DBPCFC (Table 1). All these studies recruited more than 100,000 individuals in total. Following JBI Critical Appraisal Checklist for Studies Reporting Prevalence Data, 56 of 71 studies were considered as moderate or high quality and were included for final meta-analysis (Table S2).

### 3.3. Prevalence of Wheat Allergy

In this meta-analysis, the methods were divided into self-reported (SR), self-reported physician-diagnosed (SRPD), skin prick test (SPT), specific IgE (sIgE), and food challenge (FC). The pooled prevalence of each diagnostic method was analyzed. The subgroups of region, age, basic economic condition, and timespan were also analyzed, respectively.

#### 3.3.1. SR Wheat Allergy

**Figure 2:**
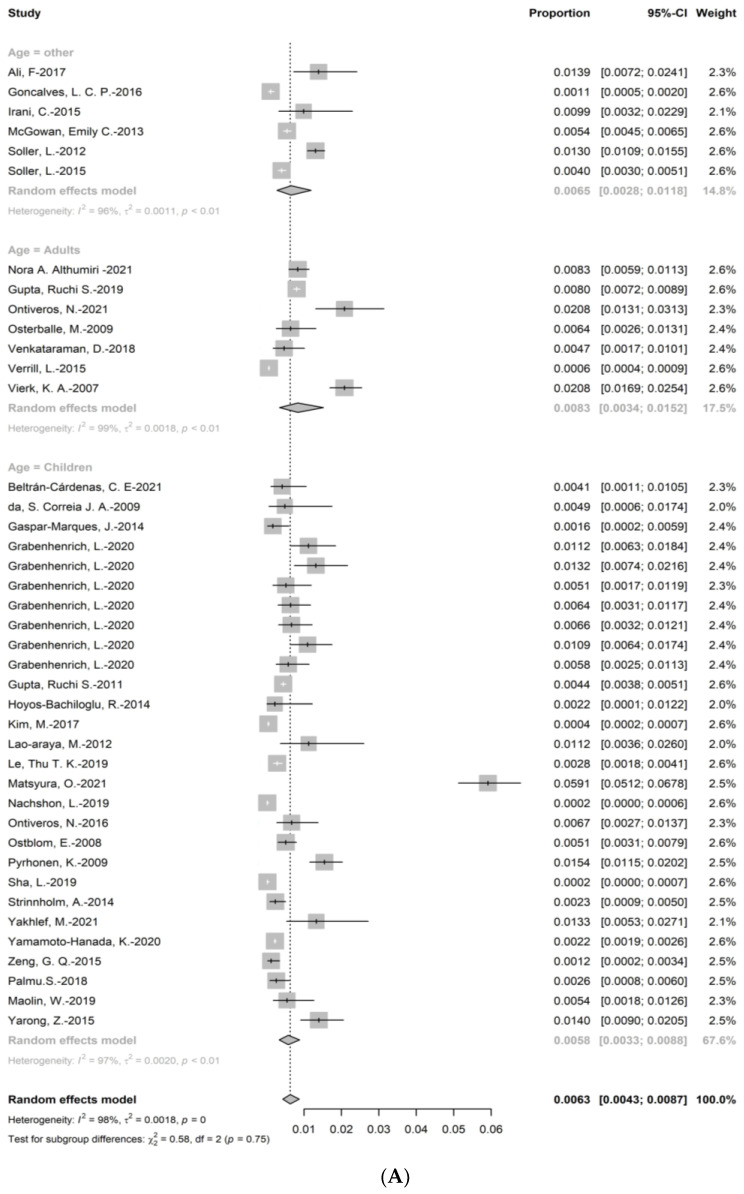

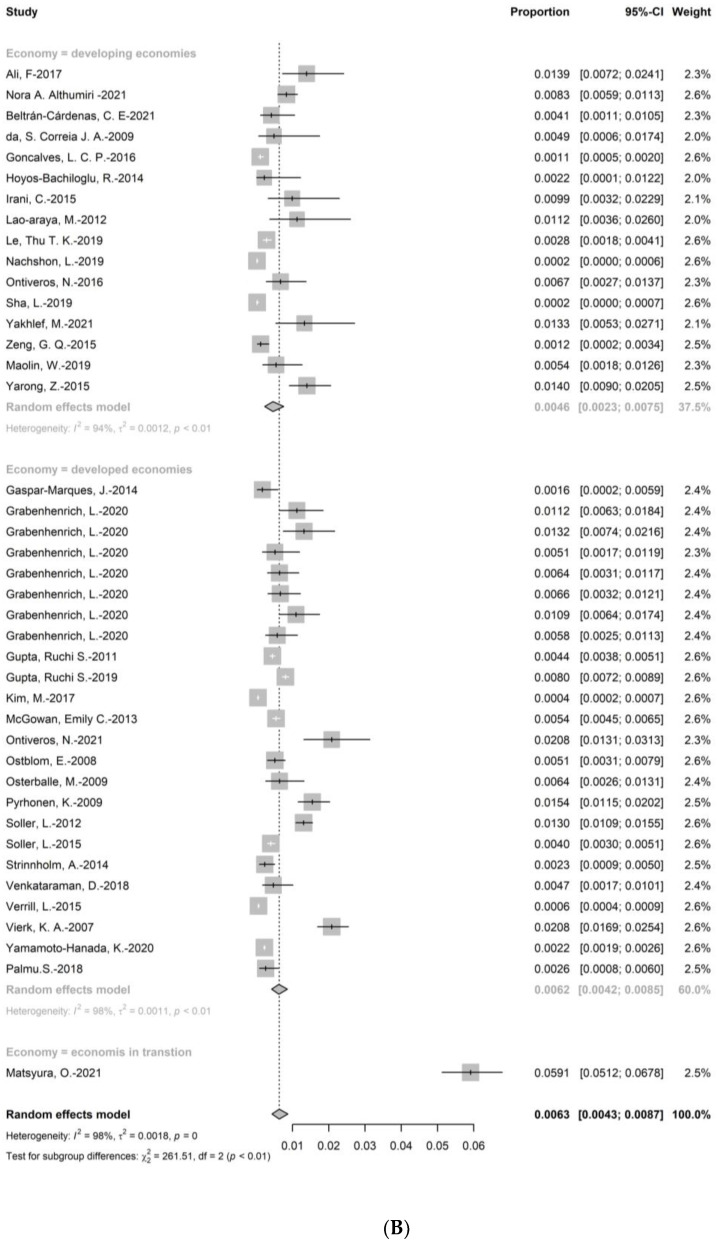

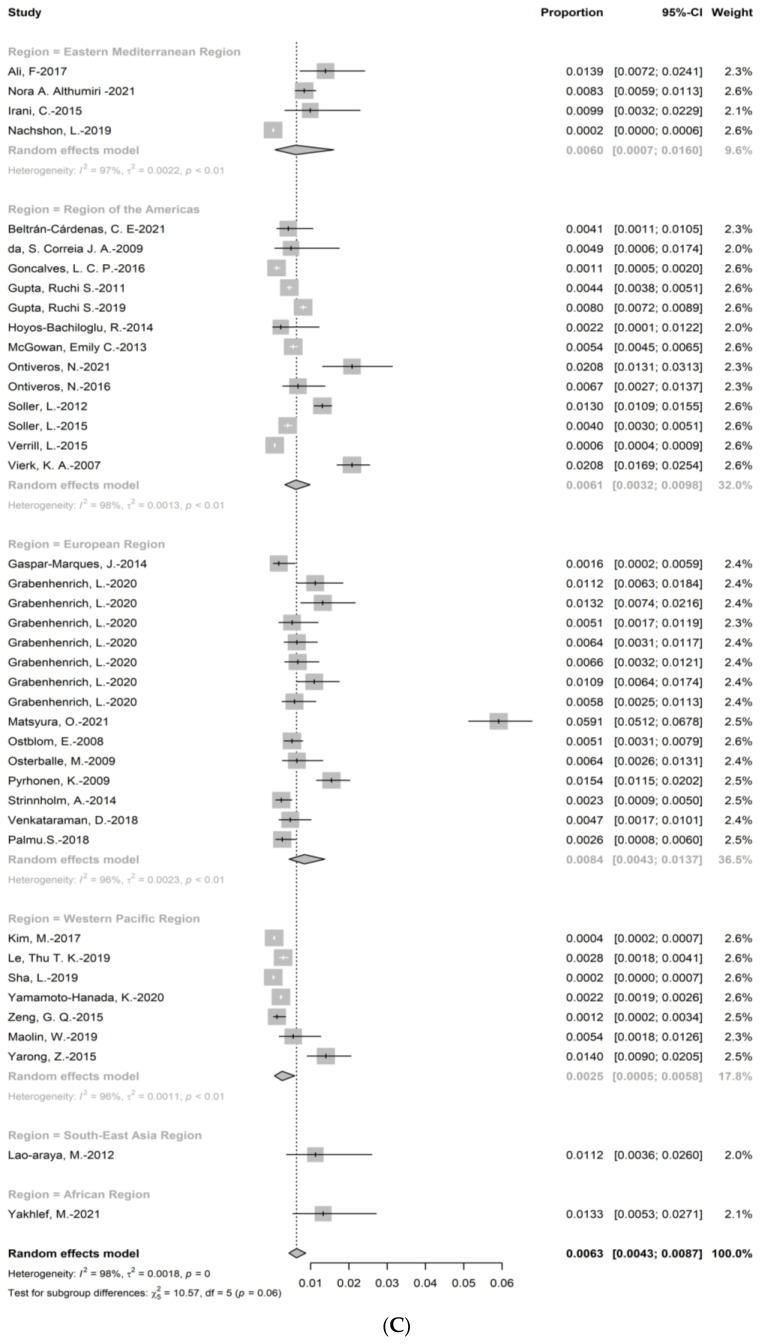

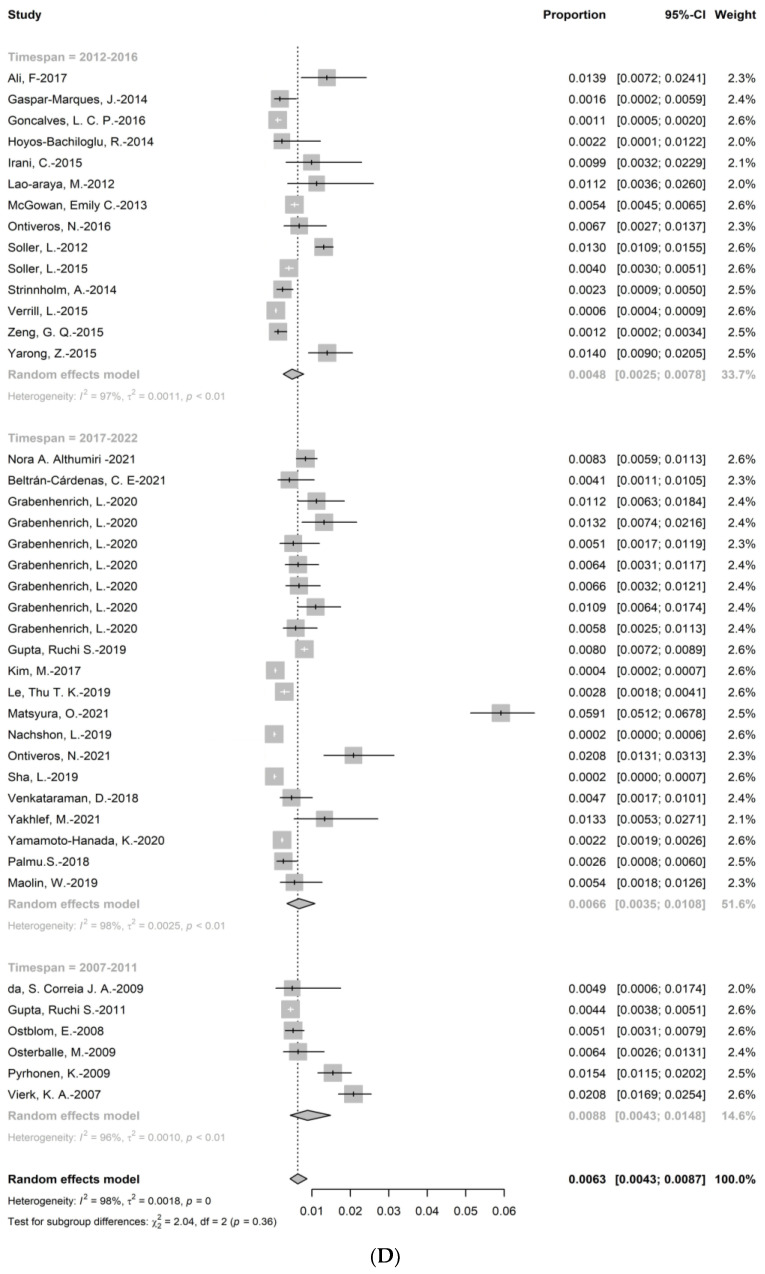
The prevalence of self-reported wheat allergy; (**A**): the subgroup analysis for age; (**B**): the subgroup analysis for economy; (**C**): the subgroup analysis for region, (**D**): the subgroup analysis for timespan [18,19,21,23,26,27,28,30,31,32,34,35,36,37,41,42,44,46,47,49,50,51,54,55,56,57,59,62,63,66,67,68,70,71,72].

Among included articles, the investigation of self-reported wheat allergy enrolled approximately 400,000 participants, in which 1676 individuals claimed they suffered from wheat allergy. The point prevalence of SR wheat allergy was 0.63% (95% CI: 0.43–0.87%) (Figure 2). The subgroup analysis showed that the prevalence of wheat allergy in adults (0.83% (95%CI: 0.34–1.52%)) was higher than in children (0.58% (95%CI: 0.33–0.88%), and the prevalence in regions with developed economic condition (0.62% (95%CI: 0.42–0.85%)) was higher than in regions with developing economic conditions (0.46% (95%CI:0.23–0.75%)). As for six regions in the world, the prevalence of wheat allergy in African regions (1.33% (95%CI: 0.53–2.71%)) was the highest, followed by South-East Asia regions (1.12% (95%CI: 0.36–2.60%), European regions (0.84% (95%CI:0.43–1.37%)), regions of Americas (0.61% (95%CI: 0.32–0.98%)), and Eastern Mediterranean regions (0.60% (95%CI: 0.07–1.60%)). Furthermore, the analysis showed that the prevalence of wheat allergy decreased over time (0.88% (95%CI: 0.43–1.48%) in timespan 1 versus 0.66% (95%CI: 0.35–1.08%) in timespan 3). The results of prevalence were stable according to sensitivity analysis, since no single study would influence the overall results in the meta-analysis (Figure S1).

#### 3.3.2. SRPD Wheat Allergy

**Figure 3:**
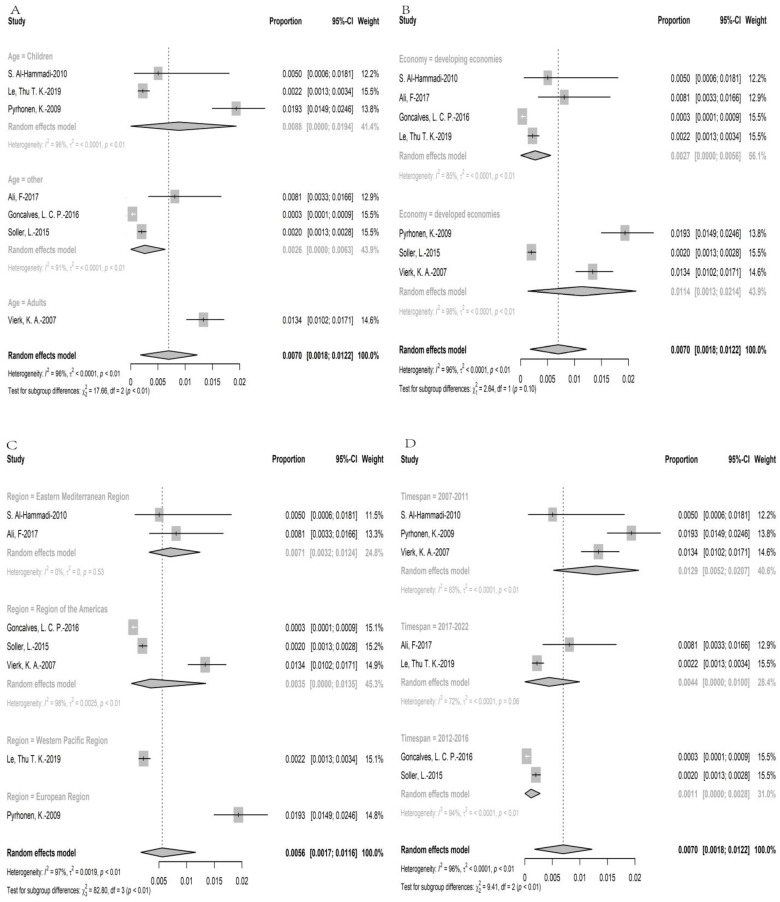
The prevalence of self-reported physician diagnosed wheat allergy; (**A**): the subgroup analysis for age; (**B**): the subgroup analysis for economy; (**C**): the subgroup analysis for region, (**D**): the subgroup analysis for timespan [17,18,27,37,51,56,63].

Among included articles, more than 40,000 participants were investigated through SRPD, in which 186 individuals claimed they suffered from wheat allergy. The point prevalence of SRPD wheat allergy was 0.70% (95% CI: 0.18–1.22%) (Figure 3). The subgroup analysis showed that the prevalence of wheat allergy in adults (1.34% (95%CI: 1.02–1.71%)) was higher than in children (0.88% (95%CI: 0–1.94%), and the prevalence in regions with developed economic condition (1.14% (95%CI: 0.13–2.14%)) was higher than in regions with developing economic condition (0.27% (95%CI:0–0.56%)). As for six regions in the world, the prevalence of wheat allergy in European regions (1.93% (95%CI: 1.49–2.46%)) was the highest, followed by Eastern Mediterranean regions (0.71% (95%CI: 0.32–1.24%)), regions of Americas (0.35% (95%CI: 0–1.35%)), and Western Pacific regions (0.22% (95%CI: 0.13–0.34%)), while South-East Asia regions had no data. Furthermore, the analysis showed that the prevalence of wheat allergy decreased over time (1.29% (95%CI: 0.52–2.07%) in timespan 1 versus 0.44% (95%CI: 0–1.00%) in timespan 3. The results of prevalence were stable according to sensitivity analysis, since no single study would influence the overall results in the meta-analysis (Figure S2).

#### 3.3.3. Wheat Allergy for SPT Positive

**Figure 4:**
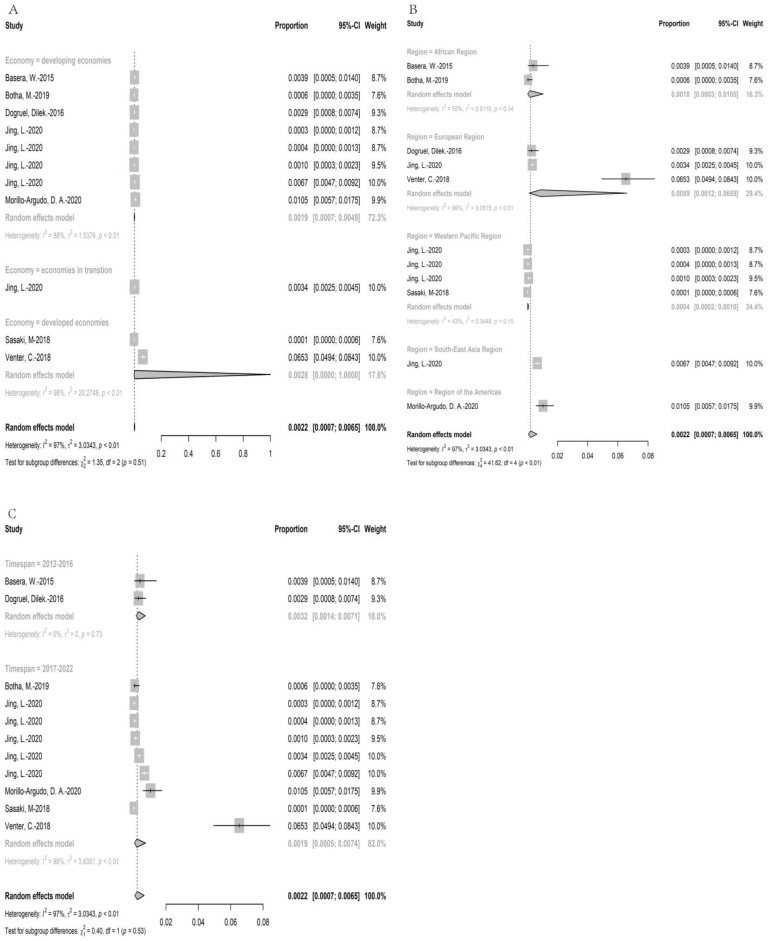
The prevalence of wheat allergy for skin prick test positive; (**A**): the subgroup analysis for economy; (**B**): the subgroup analysis for region, (**C**): the subgroup analysis for timespan [20,22,24,25,38,43,52,61].

A total of 51,656 participants were recruited for clinical examination, and the number of patients with SPT positive was 172. The point prevalence of SPT positive was 0.22% (95% CI: 0.07–0.65%) (Figure 4). On account of all participants being children, the comparison between the prevalence in children and in adults could not be achieved. The prevalence in regions with developed economic condition (0.28% (95%CI: 0–1.00%)) was higher than in regions with developing economic conditions (0.19% (95%CI:0.07–0.49%)). Moreover, the prevalence of wheat allergy in regions of Americas (1.05% (95%CI: 0.57–1.75%)) was the highest, followed by European regions (0.71% (95%CI: 0.32–1.24%)), South-East Asia regions (0.35% (95%CI: 0–1.35%)), African regions (0.22% (95%CI: 0.13–0.34%)), and Western Pacific regions (0.04% (95%CI: 0.02–0.10%)), while Eastern Mediterranean regions had no data. Furthermore, the analysis showed that the prevalence of wheat allergy decreased over time (0.32% (95%CI: 0.14–0.71%) in timespan 1 versus 0.19% (95%CI: 0.05–0.74%) in timespan 3). The results of prevalence were stable according to sensitivity analysis, since no single study would influence the overall results in the meta-analysis. (Figure S3).

#### 3.3.4. Wheat Allergy for sIgE Positive

**Figure 5:**
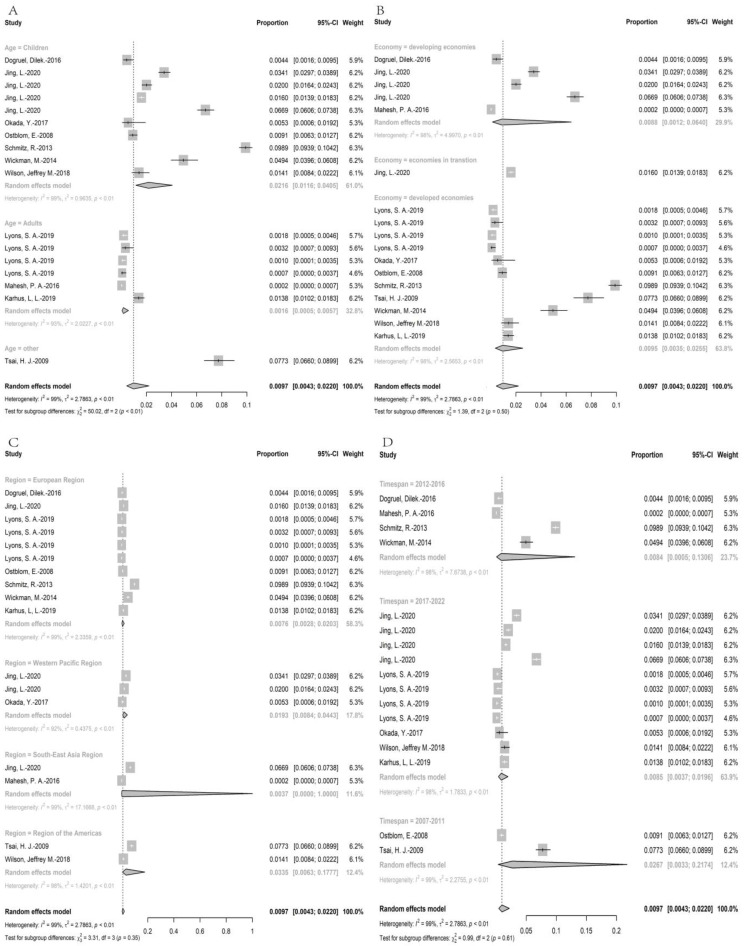
The prevalence of wheat allergy for sIgE positive; (**A**): the subgroup analysis for age; (**B**): the subgroup analysis for economy; (**C**): the subgroup analysis for region, (**D**): the subgroup analysis for timespan [25,38,39,40,45,48,53,58,64,65,69].

Approximately 75,000 individuals were recruited for wheat allergens serology test, and the individuals with sIgE-positive were 2546. The point prevalence of wheat allergy for sIgE positive was 0.97% (95% CI: 0.43–2.20%) (Figure 5). The subgroup analysis showed that the prevalence of wheat allergy in children (2.16% (95%CI: 1.16–4.05%)) was higher than in adults (0.16% (95%CI: 0.05–0.57%). The prevalence in regions with developed economic conditions (0.95% (95%CI: 0.35–2.55%)) was higher than in regions with developing economic condition (0.88% (95%CI:0.12–6.40%)). Moreover, the prevalence of wheat allergy in regions of Americas (3.35% (95%CI: 0.63–17.77%)) was the highest, followed by Western Pacific regions (1.93% (95%CI: 0.84–4.43%)), European regions (0.76% (95%CI: 0.28–2.03%)), South-East Asia regions (0.35% (95%CI: 0–1.35%)), while African regions and Eastern Mediterranean regions had no data. Furthermore, the analysis showed that the prevalence of wheat allergy decreased over time (2.67% (95%CI: 0.33–21.74%) in timespan 1 versus 0.85% (95%CI: 0.37–1.96%) in timespan 3. The results of prevalence were stable according to sensitivity analysis, since no single study would influence the overall results in the meta-analysis (Figure S4).

#### 3.3.5. Wheat Allergy Confirmed by FC

**Figure 6:**
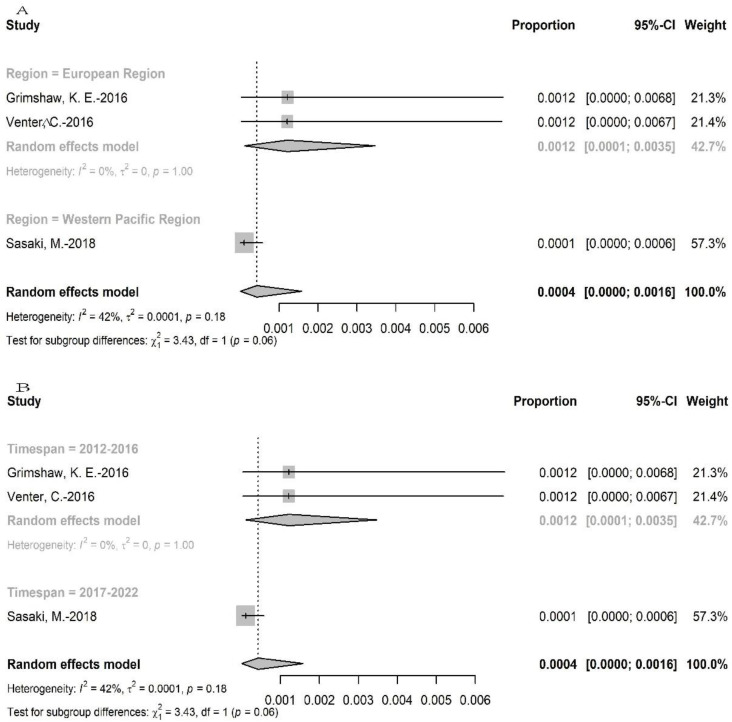
The prevalence of wheat allergy confirmed by food challenge (**A**): the subgroup analysis for region, (**B**): the subgroup analysis for timespan [29,52,60].

More than 11,000 individuals were recruited for the challenge test, and there were three allergic individuals confirmed by FC. The point prevalence of wheat allergy confirmed by FC was 0.02% (95% CI: 0–0.05%) (Figure 6). The quantities of included studies were too small to perform subgroup analysis of age and basic economic condition. The prevalence of wheat allergy was 0.12% (95%CI: 0.63–17.77% in European regions and 0.01% (95%CI: 0–0.06%) in Western Pacific regions, while no data were available for the Americas, Southeast Asia, Africa, and the Eastern Mediterranean regions. Furthermore, the analysis showed that the prevalence of wheat allergy decreased over time (0.12% (95%CI: 0.01–0.35%) in timespan 2 versus 0.01% (95%CI: 0–0.06%) in timespan 3. The results of prevalence were stable according to sensitivity analysis, since no single study would influence the overall results in the meta-analysis (Figure S5).

## 4. Discussion

This meta-analysis is indeed the first study to estimate the global prevalence of wheat allergy, even though Zuidmeer and colleagues systematically reviewed the world-wide prevalence of plant allergy, but they did not report the result of wheat allergy [73]. In this meta-analysis, we discussed the prevalence of wheat allergy under five diagnostic methods, which are SR, SRPD, SPT, sIgE, and FC. The results showed that the prevalence of wheat allergy for sIgE positive (0.97% (95%CI: 0.43–2.20%)) was the highest, followed by SRPD wheat allergy (0.70% (95% CI: 0.18–1.22%)), SR wheat allergy (0.63% (95% CI: 0.43–0.87%)), SPT positive (0.22% (95% CI: 0.07–0.65%)), and wheat allergy confirmed by FC (0.02% (95% CI: 0–0.05%)). It is notable that, in this manuscript, only three included studies were designated as FC (two in UK and one in Australia), and the FC can be largely subjective, and more prevalence data from other countries are needed to serve for the global analysis. Therefore, the prevalence of wheat allergy confirmed by FC reported in this analysis may be not entirely trustworthy. In general, the results of age subgroup analysis showed that the prevalence of wheat allergy in adults was higher than in children, except for wheat allergy for sIgE positive people (the prevalence in children was higher than in adults). However, no difference (*p* > 0.05) was found in the age subgroup of SR wheat allergy, while it was found for others. The results of basic economic condition subgroup analysis also indicated that there were more individuals suffering from wheat allergy in regions with developed economic condition than in regions with developing economic condition, but the pattern was reversed for sIgE-positive wheat allergic individuals. Additionally, the significant difference (*p* < 0.05) was only found in subgroup analysis of SR wheat allergy. When analyzing the prevalence in different geographical regions of the world, the prevalence of SR wheat allergy in African regions was the highest, while SRPD wheat allergy or wheat allergy confirmed by FC in European region was the highest, and wheat allergy for SPT or sIgE positive for regions of Americas was the highest. Moreover, subgroup analysis of timespan revealed that the prevalence of wheat allergy decreased overtime, and only the prevalence of SRPD wheat allergy was decreased significantly (*p* < 0.05).

In this meta-analysis, all steps of the literature search, screening, and comprehensive analysis followed rigorous criteria to estimate the worldwide prevalence of wheat allergy. An amount of 56 of 71 studies were eligible for inclusion in the meta-analysis after risk-of-bias assessment. Some prevalence studies included selection bias because the population for investigation came from the allergy clinic. In this meta-analysis, the design of included studies has been carefully reviewed, and the research that investigated the prevalence among patients with allergic disease was excluded to minimize the selection bias. Moreover, the language of publications was restricted to English and Chinese, and this kind of selection bias was hard to avoid due to the authors’ language skill. It is worthwhile to note that, in the survey practice, many factors would affect the findings of the prevalence of wheat allergy. As for the prevalence of SR wheat allergy, the knowledge of wheat allergy among individuals would influence the prevalence because they may misunderstand the intolerance or toxicities for allergy. Additionally, in an era when gluten-free diets remain popular, individuals with wheat allergy can avoid wheat and its products intentionally, which could lead to a bias in the prevalence of wheat allergy. However, the cross-contact of gluten during the processing of pre-packaged food could induce an unintended wheat allergy, preventing consumers from realizing the cause of allergic reaction. Thus, the prevalence of SR wheat allergy would be misleading. Wheat allergy for SPT or sIgE positive would also sometimes be affected due to the inaccuracy of self-reported clinical history. Additionally, diagnostic criteria in the included studies were inconsistent when making the diagnosis of wheat allergy, such as the size of the wheal for SPT or the cut-off value of sIgE for blood test, hence the point prevalence of wheat allergy in this study needs to be interpreted with caution. Additionally, the cross-reaction between wheat and grass or other cereals would lead to a SPT or sIgE false positive result for wheat allergy [74]. Moreover, the WHO/FAO ad hoc joint expert group divided the quality of IgE-mediated food allergy prevalence data into three grades [75], and GRADE 1 was the combination of clinical reaction, evidence of sensitization, and food challenge. GRADE 2 was adverse symptoms, together with the evidence of biomarkers, such as sensitization identified by SPT or sIgE; GRADE 3 came from the data about self-reported results alone, as well as only evidence of IgE data to identify food allergy, and patients with allergy were identified by retrospective review of medical records. Therefore, FC is the most convincing method to diagnose wheat allergy. Although DBPCFC is the “Golden Standard” for diagnosing, open FC or single blind FC is also an appropriate method, since there were still many challenges and limitations in DBPCFC practice.

Better awareness of the characteristics would be achieved by subgroup analysis. In general, the results of age subgroup analysis showed that the prevalence of wheat allergy in adults’ subgroup was higher than in children, except for wheat allergy for those that are sIgE positive. However, no difference (*p* > 0.05) was found in the age subgroup of SR wheat allergy, while others were found. Siripipattanamongkol, N et al. [76]. found that the proportion of wheat-tolerant children increased over time. Christensen, MJ et al. [77]. showed that the vast majority of younger children can develop tolerance, whereas elder children and adults rarely develop tolerance, which would explain the higher prevalence of wheat allergy in adults in this analysis. According to criteria for quality evaluation of prevalence data recommended by ad hoc joint FAO/WHO expert consultation, low quality prevalence data (SR or SRPD) in this meta-analysis indicated a higher prevalence of wheat allergy in adults than in children, and medium quality prevalence data (sIgE or SPT with clinical history) indicated a higher prevalence in children than in adults. Therefore, high quality of prevalence data (FC) is needed to investigate the real situation of wheat allergy. Moreover, the reasons for such a difference deserve further investigation.

The results of subgroup analysis also indicated that there were more individuals suffering from wheat allergy in regions with developed economic condition than in regions with developing economic condition. Additionally, only the prevalence of SR in regions with developed economic condition was significantly higher (*p* < 0.05) than in regions with developing economic condition. Although the present analysis based on reported data suggested a lower prevalence of wheat allergy in countries with developing economic conditions, fewer studies about countries with developing economic condition were included for analysis than studies about countries with developed economic condition, so the results need to be treated with caution. Lee, KS et al. [78]. found that high socioeconomic status was a risk factor for allergic diseases in Korean adolescents, and people with high socioeconomic status had access to better medical care, which not only increased the diagnosis of allergic diseases, but may also influence the prevalence of allergic diseases due to various immunizations.

When analyzing the prevalence in different geographical regions of the world, the prevalence of SR wheat allergy in African regions was the highest, while SRPD wheat allergy or wheat allergy confirmed by FC in European region was the highest, wheat allergy for SPT or sIgE positive for regions of Americas was the highest, and significant difference (*p* < 0.05) was only found in subgroup analysis of SRPD wheat allergy and wheat allergy for SPT positive. The low altitude of the European region, its high latitude in the Northern Hemisphere, and the fact that the diet is mostly Mediterranean, where grains would be the main source of daily food, could be potential reasons for the higher prevalence.

Moreover, subgroup analysis of timespan revealed that the prevalence of wheat allergy decreased overtime, but no significant difference (*p* > 0.05) was found, except for SRPD wheat allergy. Compared to the prevalence of wheat allergy in Europe reported by Nwaru, B.I., et al. [79], the prevalence summarized in this meta-analysis was lower from the perspective of SR, SRPD, SPT, and sIgE. The reason for this difference may be the basic economic condition, geographical location, genetic factor, etc. As is shown in this manuscript, the prevalence of wheat allergy decreased overtime, and this is probably because people are more cautious in recognizing allergies with the popularization of knowledge about allergies in recent years, and the clinical diagnosis became more standard. Moreover, the study design, as well as characteristics of population and diagnostic criteria, may also influence the result of prevalence in different time.

## 5. Conclusions

The present meta-analysis indicated the prevalence of wheat allergy was about 0.63% for SR, 0.70% for SRPD, 0.22% for SPT positive to wheat allergens, 0.97% for sIgE positive, and 0.04% for FC-confirmed. The age, basic economic condition, or geographical region could be the factors that influence the prevalence of wheat allergy, and they are worthwhile for further investigation. The DBPCFC is the ‘Gold Standard’ for diagnosing food allergy [80]. The included studies in this meta-analysis were rarely used for food challenges to diagnose wheat allergy, and they would lead to the prevalence of wheat allergies in this analysis, deviating from the real world. Future studies about the prevalence of wheat allergy should promote multi-regional or multi-national collaborative research through consistent criteria and diagnostic methods, which can reduce the bias caused by study designs or diagnostic methods. Investigating the prevalence of wheat allergies in the real world as accurately as possible will contribute to the prevention, management, and risk assessment of wheat allergy.

## Figures and Tables

**Table 1:** Summary of the characteristics of 56 included articles.

No	First Author, Published Year	Study Design	Study Period (Year, or Year and Month)	Sample Size	Country	Age of Population	Diagnostic Method *
1	S. Al-Hammadi., 2010 [17]	Cross-sectional study	December 2006	397	The United Arab Emirates	Children	SRPD
2	Ali, F., 2017 [18]	Cross-sectional study	May 2015–January 2015	865	Kuwait	Other	SR SRPD
3	Nora A. Althumiri., 2021 [19]	Cross-sectional study	January 2020	4709	Saudi Arabia	Adults	SR
4	Basera, W., 2015 [20]	Cross-sectional study	February 2013–December 2014	512	South Africa	Children	SPT
5	Beltrán-Cárdenas, C.E., 2021 [21]	Cross-sectional study	August 2019–September 2019	969	Colombia	Children	SR
6	Botha, M., 2019 [22]	Cross-sectional study	February 2013–December 2016	1583	South Africa	Children	SPT
7	da, S. Correia, J.A., 2009 [23]	Cross-sectional study	2019–2020	412	Brazil	Children	SR
8	Dean, T., 2007 [24]	Cohort study	September 2001–August 2002	807	UK	Children	SPT
9	Dogruel, D., 2016 [25]	Cohort study	February 2010–February 2011	1377	Turkey	Children	SPT sIgE
10	Gaspar-Marques, J., 2014 [26]	Cross-sectional study	NA	1217	Portugal	Children	SR
11	Goncalves, L.C.P., 2016 [27]	Cross-sectional study	March 2012–September 2013	9265	Brazil	Other	SR SRPD
12	Grabenhenrich, L., 2020 [28]	Cohort study	2013–2017	1341 1140 976 1570 1513 1556 1387	Iceland UK Netherland Germany Poland Lithuania Spain	Children	SR
13	Grimshaw, K.E., 2016 [29]	Cohort study	2006–2008	823	UK	Children	FC
14	Gupta, Ruchi S., 2011 [30]	Cross-sectional study	June 2009–February 2010	38,480	US	Children	SR
15	Gupta, Ruchi S., 2019 [31]	Cross-sectional study	October 2015–September 2016	40,443	US	Adults	SR
16	Hoyos-Bachiloglu, R., 2014 [32]	Cross-sectional study	September 2011–December 2012	455	Chile	Children	SR
17	Yan, H., 2010 [33]	Well health check	2009	382	China	Children	SPT
18	Irani, C., 2015 [34]	Cross-sectional study	July 2014 + 7 weeks	506	Lebanon	Other	SR
19	Kim, M., 2017 [35]	Cross-sectional study	September 2015	29,842	Korea	Children	SR
20	Lao-araya, M., 2012 [36]	Cross-sectional study	2010	446	Thailand	Children	SR
21	Le, Thu T.K., 2019 [37]	Cross-sectional study	2016	8620	Vietnam	Children	SR SRPD
22	Jing, L., 2020 [38]	Cross-sectional study	September 2009–June 2016	6194 5139 12,997 5677	China China Russia India	Children	SPT sIgE
23	Lyons, S.A., 2019 [39]	Cross-sectional study	2005–2009	2229 935 2078 1497	Sweden Spain Iceland Poland	Adults	sIgE
24	Mahesh, P.A., 2016 [40]	Cross-sectional study	2005–2009	10,931	India	Adults	sIgE
25	Matsyura, O., 2021 [41]	Cross-sectional study	2016–2017	935	Ukrine	Children	SR
26	McGowan, Emily C., 2013 [42]	Cross-sectional study	2007–2010	2078	US	Other	SR
27	Morillo-Argudo, D.A., 2020 [43]	Cross-sectional study	July 2013–July 2014	1497	Ecuador	Children	SPT
28	Nachshon, L., 2019 [44]	Cross-sectional study	May-October 2016	12,592	Israel	Children	SR
29	Okada, Y., 2017 [45]	Cross-sectional study	July 2014–February 2015	374	Japan	Children	sIgE
30	Ontiveros, N., 2021 [46]	Cross-sectional study	October 2020	1058	Paraguayan	Adults	SR
31	Ontiveros, N., 2016 [47]	Cross-sectional study	September 2014–August 2015	1049	Mexico	Children	SR
32	Ostblom, E., 2008 [48]	Cohort study	1994–1996	3742	Sweden	Children	sIgE
33	Ostblom, E., 2008 [49]	Cohort study	1994–1996	3742	Sweden	Children	SR
34	Osterballe, M., 2009 [50]	Cross-sectional study	NA	1094	Denmark	Adults	SR
35	Pyrhonen, K., 2009 [51]	Cross-sectional study	April 2001–March 2005	3308	Finland	Children	SR SRPD
36	Sasaki, M., 2018 [52]	Cross-sectional study	2011–2014	9663	Australia	Children	FC
37	Schmitz, R., 2013 [53]	Cross-sectional study	May 2003–May 2006	12,998	Germany	Children	sIgE
38	Sha, L., 2019 [54]	Cross-sectional study	2010	13,073	China	Children	SR
39	Soller, L., 2012 [55]	Cross-sectional study	May 2008–March 2009	9667	Canada	Other	SR
40	Soller, L., 2015 [56]	Cross-sectional study	2010–2011	15,022	Canada	Other	SR SRPD
41	Strinnholm, A., 2014 [57]	Cohort study	2006–2010	2585	Sweden	Children	SR
42	Tsai, H.J., 2009 [58]	Cross-sectional study	August 2005–May 2008	2004	US	Other	sIgE
43	Venkataraman, D., 2018 [59]	Cohort study	2007	1290	UK	Adults	SR
44	Venter, C., 2016 [60]	Cohort study	2011–2012	827	UK	Children	FC
45	Venter, C., 2018 [61]	Cohort study	2011–2012	827	UK	Children	SPT
46	Verrill, L., 2015 [62]	Cross-sectional study	2007–2010	44,778	US	Adults	SR SRPD
47	Vierk, K.A., 2007 [63]	Cross-sectional study	April 2001–August 2011	4568	US	Adults	SR SRPD
48	Wickman, M., 2014 [64]	Cohort study	2010–2012	1699	Sweden	Children	sIgE
49	Wilson, J.M., 2018 [65]	Cohort study	1999–2002	1279	US	Children	sIgE
50	Yakhlef, M., 2021 [66]	Cross-sectional study	15 April 2018–29 April 2018	528	Algeria	Children	SR
51	Yamamoto-Hanada, K., 2020 [67]	Cross-sectional study	January 2011–March 2014	92,945	Japan	Children	SR
52	Zeng, G.Q., 2015 [68]	Cross-sectional study	January 2013–December 2013	2540	China	Children	SR
53	Karhus, L, L., 2019 [69]	Cross-sectional study	June 2006–June 2008	3405	Denmark	Adults	sIgE
54	Palmu.S., 2018 [70]	Cross-sectional study	May 2016–September 2016	1937	Finland	Children	SR
55	Maolin, W., 2019 [71]	Cross-sectional study	March 2014–March 2015	923	China	Children	SR
56	Yarong, Z., 2015 [72]	Cross-sectional study	November 2011–April 2012	1792	China	Children	SR

* SR: self-reported; SRPD: self-reported physician diagnosed; SPT: skin prick test; sIgE: specific immunoglobulin-E; FC: food challenge; DBPCFC: double-blind placebo-controlled food challenge.

## Data Availability

Data is contained within the article or supplementary material.

## Supplementary Materials

The following supporting information can be downloaded at: https://www.mdpi.com/article/10.3390/nu15071564/s1, Figure S1: Sensitivity analysis of studies for self-reported wheat allergy, Figure S2: Sensitivity analysis of studies for self-reported physician-diagnosed wheat allergy, Figure S3: Sensitivity analysis of studies of wheat allergy for SPT positive to wheat allergens, Figure S4: Sensitivity analysis of studies of wheat allergy for sIgE positive to wheat allergens, Figure S5: Sensitivity analysis of studies for food challenge confirmed wheat allergy, Table S1: Joanna Briggs Institute Critical Appraisal Checklist for Studies Reporting Prevalence Data, Table S2: The results of risk bias assessment (Y: Yes; N: No;).
