# Supplementary material for: A Meta-Analysis of the Prevalence of Wheat Allergy Worldwide
Source: Nutrients. 2023 Mar 23;15(7):1564. doi: 10.3390/nu15071564 (PMC10097276; doi:10.3390/nu15071564)
Supplement: Supplementary file 1 [file nutrients-15-01564-s001.zip › nutrients-2256102-supplementary.pdf]

Table S1 Joanna Briggs Institute Critical Appraisal Checklist for Studies Reporting Prevalence Data.

	Yes	No	Unclear
1. Was the sample frame appropriate to address the target population?	☐	☐	☐
2. Were study participants sampled in an appropriate way?	☐	☐	☐
3. Was the sample size adequate?	☐	☐	☐
4. Were the study subjects and the setting described in detail?	☐	☐	☐
5. Was the data analysis conducted with sufficient coverage of the identified sample?	☐	☐	☐
6. Were valid methods used for the identification of the condition?	☐	☐	☐
7. Was the condition measured in a standard, reliable way for all participants?	☐	☐	☐
8. Was there appropriate statistical analysis?	☐	☐	☐
9. Was the response rate adequate, and if not, was the low response rate managed appropriately?	☐	☐	☐

Table S2. The results of risk bias assessment (Y: Yes; N: No;).

Author	Year	Q1*	Q2	Q3	Q4	Q5	Q6	Q7	Q8	Q9	Overall appraisal
S. Al-Hammadi.	2010	Y	Y	Y	Y	Y	Y	Unclear	Unclear	N	Include
Ali, F.	2017	Y	Y	Unclear	Y	Y	Y	Unclear	Unclear	Y	Include
Allen, Katrina J.	2009	Y	Y	Y	N	Y	Unclear	Unclear	Unclear	N	Exclude
Aramburo-Galvez, J. G.	2020	Unclear	N	Unclear	Y	Y	Y	Y	Y	Unclear	Exclude
Basera, W.	2015	Y	Y	Unclear	Y	Y	Y	Y	Unclear	Unclear	Include
Beltran-Cardenas, C. E.	2021	Y	Y	Y	Y	Y	Y	Unclear	Unclear	Y	Include
Botha, M.	2019	Y	Y	Y	Y	Y	Y	Y	Y	Y	Include
Cabrera-Chavez, F.	2017	Unclear	N	Unclear	Y	Y	Y	Y	Y	Unclear	Exclude
Cabrera-Chavez, F.	2016	Unclear	N	Unclear	Y	Y	Y	Y	Y	Unclear	Exclude
Cabrera-Chavez, F.	2018	Unclear	N	Unclear	Y	Y	Y	Y	Y	Unclear	Exclude
Caffarelli, C.	2011	Unclear	N	Unclear	Y	Y	Y	Y	Y	Unclear	Exclude

da, S. Correia J. A.	2009	Y	Y	Y	Y	Y	Unclear	Unclear	Y	Y	Include
Dean, T.	2007	Y	Y	Y	Y	Y	Y	Y	Unclear	Y	Include
Dogruel, D.	2016	Y	Y	Y	Y	Y	Y	Y	Unclear	Y	Include
Gaspar-Marques, J.	2014	Y	Y	Unclear	Y	Y	Y	Unclear	Unclear	Unclear	Include
Goncalves, LCP.	2016	Y	Y	Y	Y	Y	Y	Y	Unclear	Unclear	Include
Grabenhenrich, L.	2020	Y	Y	Y	Y	Y	Y	Y	Y	Y	Include
Grimshaw, K. E.	2016	Y	Y	Y	Y	Y	Y	Unclear	Unclear	Y	Include
Gupta, Ruchi S.	2011	Y	Y	Y	Y	Y	Y	Unclear	Y	Y	Include
Gupta, Ruchi S.	2019	Y	Y	Y	Y	Y	Y	Unclear	Y	Y	Include
Hoyos-Bachiloglu, R.	2014	Unclear	Y	Unclear	Y	Y	Y	Unclear	Y	Y	Include
Irani, C.	2015	Y	Y	Y	Y	Y	Unclear	Y	Unclear	Y	Include
Jarvenpaa, J.	2014	Y	Y	Unclear	Y	Y	Unclear	Unclear	Unclear	Unclear	Exclude
Jing, L.	2020	Unclear	Unclear	Y	Y	Y	Y	Y	Y	Y	Include
Kallio, P.	2011	Y	Y	Unclear	Y	Y	Unclear	Unclear	Unclear	Unclear	Exclude

Karhus, L, L.	2019	Unclear	Y	Unclear	Y	Y	Y	Y	Unclear	Unclear	Include
Kim, M.	2017	Y	Y	Y	Y	Unclear	Y	Unclear	Y	Y	Include
Lao-araya, M.	2012	Unclear	Unclear	Y	Y	Y	Y	Y	Unclear	Y	Include
Le, Thu T. K.	2019	Y	Y	Y	Y	Y	Y	Unclear	Unclear	Y	Include
Le, Thu T. K.	2018	Unclear	Unclear	Unclear	Y	Unclear	Y	Unclear	Y	Unclear	Exclude
Lyons, S. A.	2019	Y	Y	Y	Y	Y	Y	Y	Y	Y	Include
Mahesh, P. A.	2016	Y	Y	Y	Y	Y	Y	Y	Y	Y	Include
Maolin, W.	2019	Unclear	Unclear	Unclear	Y	Y	Y	Y	Unclear	Y	Include
Matsyura, O.	2021	Y	Y	Y	Y	Y	Unclear	Unclear	Unclear	Y	Include
McGowan, Emily C.	2013	Y	Y	Y	Y	Y	Y	Y	Y	Y	Include
Morillo-Argudo, D. A.	2020	Y	Y	Y	Y	Unclear	Y	Unclear	Unclear	Y	Include
Morita, E.	2012	Unclear	Unclear	Unclear	Y	Y	Y	Y	N	Unclear	Exclude
Nachshon, L.	2019	Y	Y	Y	Y	Y	Y	Y	Unclear	Y	Include
Nora A. Althumiri.	2021	Y	Y	Y	Y	Y	Unclear	Unclear	Y	Y	Include

Okada, Y.	2017	Y	Y	Unclear	Y	Y	Y	Y	Unclear	Y	Include
Ontiveros, N.	2021	Unclear	Unclear	Y	Y	Y	Y	Unclear	Unclear	Y	Include
Ontiveros, N.	2016	Unclear	Unclear	Y	Y	Y	Y	Unclear	Unclear	Y	Include
Ontiveros, N.	2018	Unclear	N	Unclear	Y	Y	Y	Y	Y	Unclear	Exclude
Ontiveros, N.	2015	Unclear	N	Unclear	Y	Y	Y	Y	Y	Unclear	Exclude
Ostblom, E.	2008	Y	Y	Unclear	Y	Y	Y	Y	Unclear	Y	Include
Ostblom, E.	2008	Y	Y	Unclear	Y	Y	Y	Y	Unclear	Y	Include
Osterballe, M.	2009	Y	Y	Unclear	Y	Y	Y	Y	N	Unclear	Include
Palmu.S.	2018	Y	Y	Y	Y	Y	Unclear	Unclear	Unclear	Y	Include
Pyrhonen, K.	2009	Y	Y	Y	Y	Y	Y	Y	Unclear	Unclear	Include
Qingqing, Y.	2019	Y	Y	Unclear	Unclear	Y	Unclear	Unclear	Unclear	Y	Exclude
Sasaki, M.	2018	Y	Y	Y	Y	Y	Y	Y	Y	Unclear	Include
Schmitz, R.	2013	Y	Y	Y	Y	Y	Y	Y	Y	Y	Include
Sha, L.	2019	Y	Y	Y	Y	Y	Y	Y	Unclear	Y	Include

Soller, L.	2012	Y	Y	Unclear	Y	Y	Y	Unclear	Y	Y	Include
Soller, L.	2015	Y	Y	Unclear	Y	Y	Y	Unclear	Y	Y	Include
Strinnholm, A.	2014	Unclear	Y	Unclear	Y	Y	Y	Y	Unclear	Unclear	Include
Taylor-Black, Sarah A.	2014	Unclear	Unclear	Unclear	Y	Y	Y	Unclear	Unclear	Y	Exclude
Tsai, H. J.	2009	Y	Y	Y	Y	Y	Y	Y	Unclear	Unclear	Include
Venkataraman, D.	2018	Y	Y	Unclear	Y	Y	Y	Y	Unclear	Unclear	Include
Venter, C.	2016	Y	Y	Unclear	Y	Y	Y	Y	Unclear	Y	Include
Venter, C.	2018	Y	Y	Unclear	Y	Y	Y	Y	Unclear	Y	Include
Verrill, L.	2015	Y	Y	Y	Y	Y	Y	Unclear	N	Unclear	Include
Vierk, K. A.	2007	Y	Y	Unclear	Y	Y	Y	Y	Y	Y	Include
Vu, N. T.	2015	Unclear	Unclear	Unclear	Unclear	Y	Y	Y	Y	Unclear	Exclude
Wickman, M.	2014	Y	Y	Unclear	Y	Y	Y	Y	Unclear	Unclear	Include
Wilson, Jeffrey M.	2018	Y	Y	Unclear	Y	Y	Y	Y	Unclear	Unclear	Include
Yakhlef, M.	2021	Y	Y	Y	Y	Y	Unclear	Unclear	Unclear	Y	Include

Yamamoto-Hanada, K.	2020	Y	Y	Y	Y	Y	Y	Y	Unclear	Y	Include
Yan, H.	2010	Unclear	Unclear	Unclear	Y	Y	Y	Y	Unclear	Y	Include
Yarong, Z.	2015	Y	Y	Unclear	Y	Y	Unclear	Y	Unclear	Y	Include
Zeng, G. Q.	2015	Y	Y	Unclear	Y	Y	Y	Y	Unclear	Y	Include

*: The details of these question (Q1-Q9) were showed in Tbale S1.

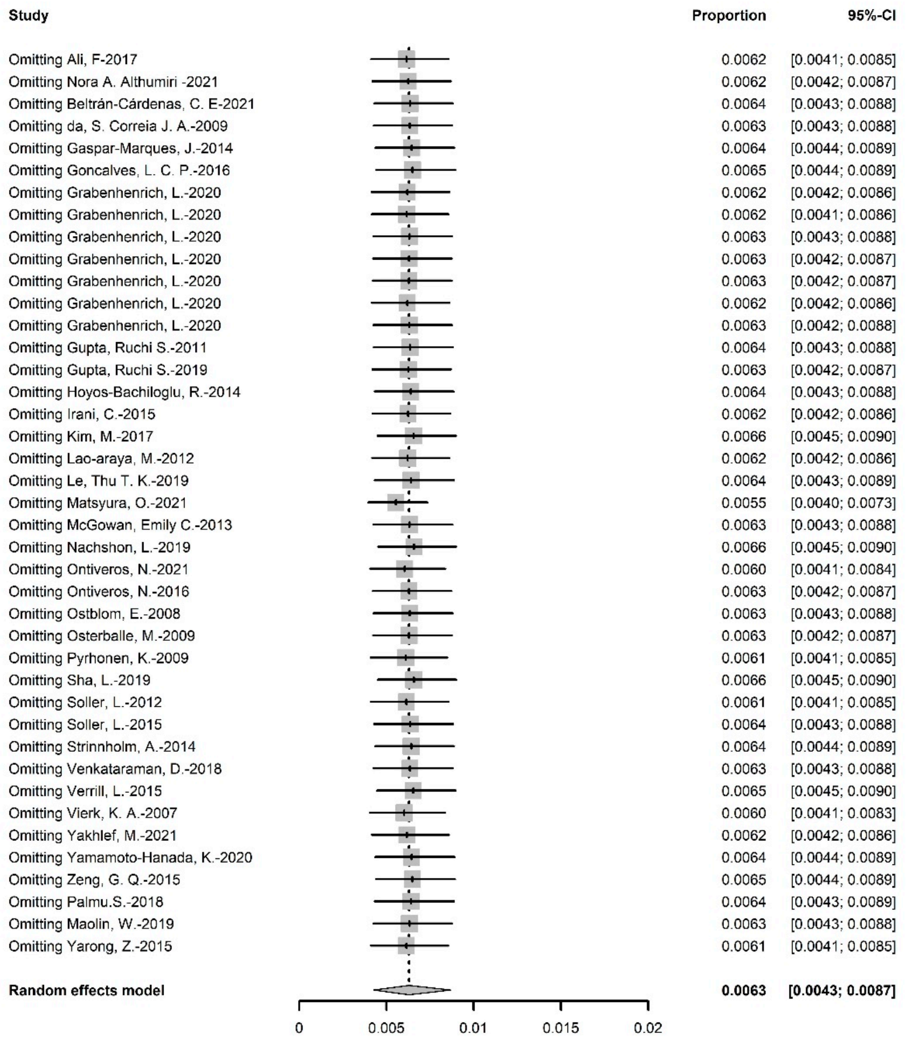

Figure S1 Sensitivity analysis of studies for self-reported wheat allergy.

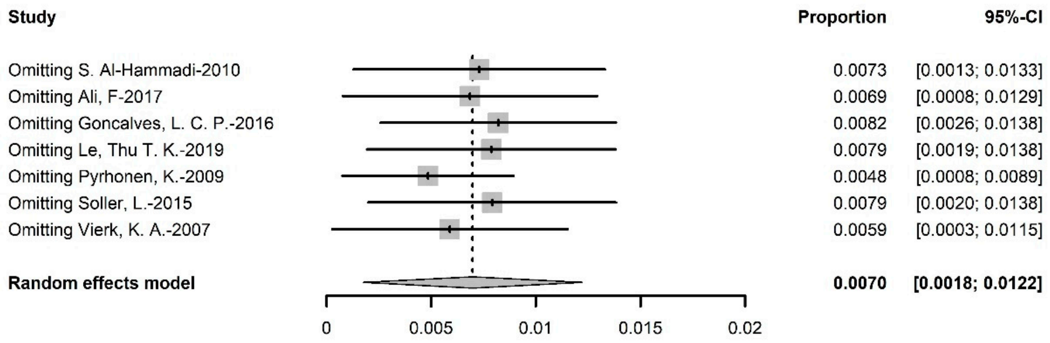

Figure S2 Sensitivity analysis of studies for self-reported physician-diagnosed wheat allergy.

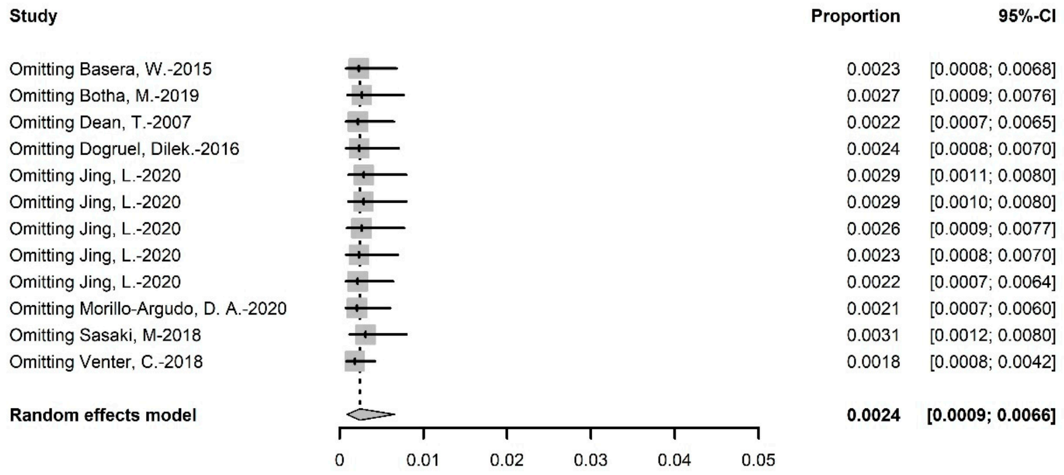

Figure S3 Sensitivity analysis of studies of wheat allergy for SPT positive to wheat allergens.

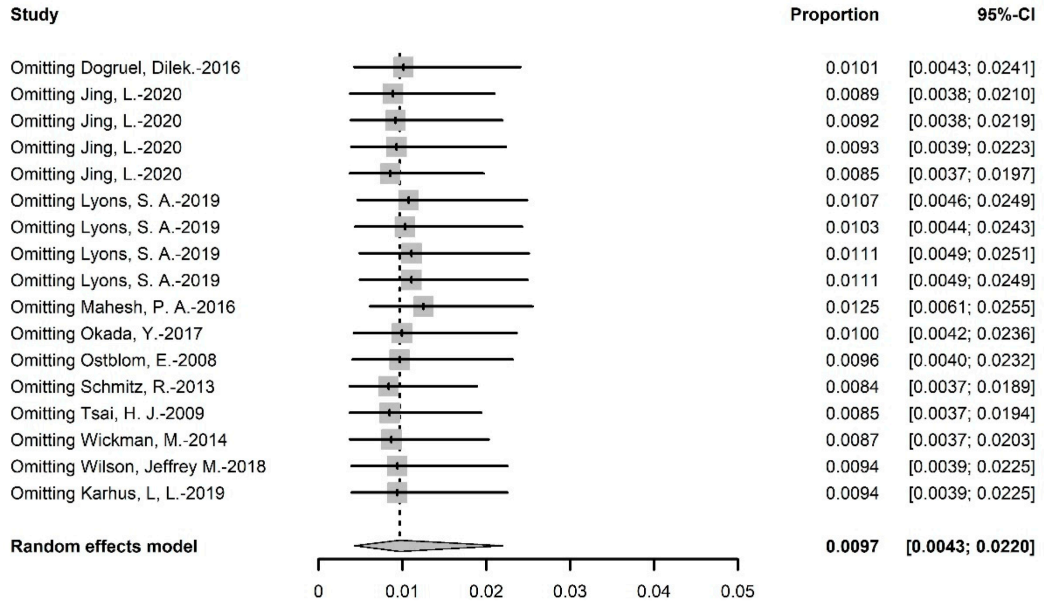

Figure S4 Sensitivity analysis of studies of wheat allergy for sIgE positive to wheat allergens.

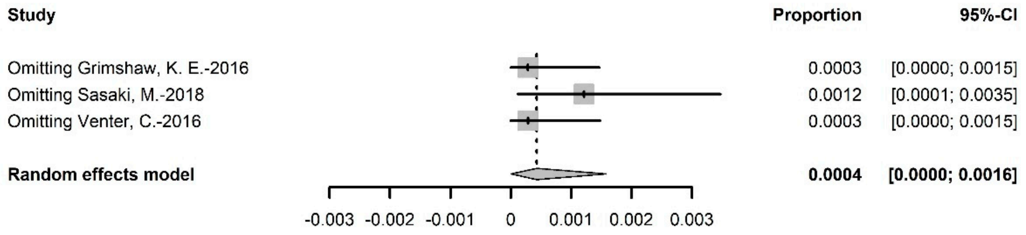

Figure S5 Sensitivity analysis of studies for food challenge confirmed wheat allergy.
